# Melanoma in situ of penis: a very rare entity: A case report and review of the literature

**DOI:** 10.1097/MD.0000000000007652

**Published:** 2017-09-08

**Authors:** Roberto Baraziol, Mauro Schiavon, Eugenio Fraccalanza, Gioacchino De Giorgi

**Affiliations:** a Azienda Sanitaria Universitaria Integrata di Udine, Plastic Surgery Unit; b Azienda Sanitaria Universitaria Integrata di Udine, Urology Unit, Udine, Italy.

**Keywords:** melanoma in situ, penis, plastic surgery

## Abstract

**Rationale:**

Melanoma in situ of the penis is very rare and there are no clear guidelines for its surgical treatment.

**Patient concerns:**

The authors describe the case of a 69-year-old man who presented with an asymptomatic brown macula on his glans penis and foreskin that appeared about 8 years earlier, enlarged in the last few months.

**Diagnoses:**

A diagnostic biopsy showed the characteristics of a melanoma in situ.

**Interventions:**

The authors decided to excise the lesion keeping a healthy margin of 1 cm all over around except close to the urethral meatus, where it was impossible, and where only 5 mm of free margin was excised. A full thickness mucosal graft from oral cavity was performed to repair the defect.

**Outcomes:**

No recurrence or metastasis occurred during 50 months after the operation.

**Lessons:**

Considering that at the sixth clinical follow-up the patient was alive and disease free at 50 months after surgery, the chosen treatment has proved successful.

## 1 Introduction

Primary penile and male urethra melanoma is a rare malignant neoplasm that mostly affects elderly patients, from the sixth and seventh decades of life.^[1]^ In literature, approximately 200 cases were described and they represented less than 1.4% of primary carcinomas of the penis^[1,2]^ and 0.1% to 0.2% of all extraocular melanomas.^[3]^ Most frequently, the lesion is located on the glans (55%), followed by foreskin (28%), penile shaft (9%) and urethral meatus (8%).^[1,4]^ Melanoma in situ (MIS) of the penis is even more rare.^[5,6]^ There is lack of consensus on the extent of treatment that is indicated.^[6]^ The authors present the ninth case of MIS of penis published in western literature and treated following the usual excision margins for a MIS in other sites.

## 2 Case report

**Figure 1:**
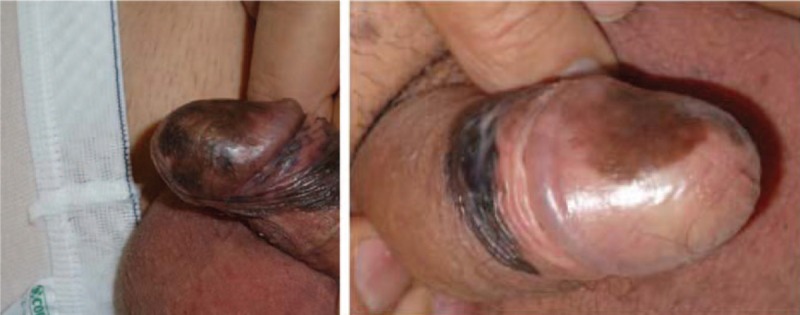
Clinical photographs of the pigmented macula on the glans and foreskin.

**Figure 2:**
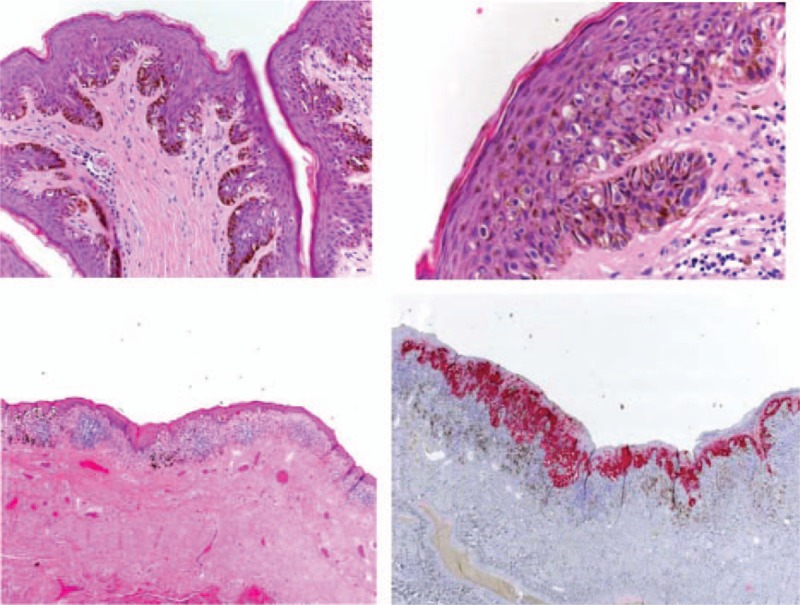
Histologic appearance showing numerous atypical melanocytic cells with large hyperchromatic nuclei and abundant cytoplasm. No dermal invasion of atypical melanocytes was seen.

A 69-year-old Caucasian man presented with a dark-brown to black pigmented macula on the glans and foreskin of several years duration. The asymptomatic pigmented lesion had rapidly enlarged in the last few months (Fig. 1). The patient came to the plastic surgical team after that the urologists performed excision of the foreskin and some biopsies on the glans to made the diagnosis of the lesion. Histological examination showed a diagnosis of MIS (Fig. 2).

**Figure 3:**
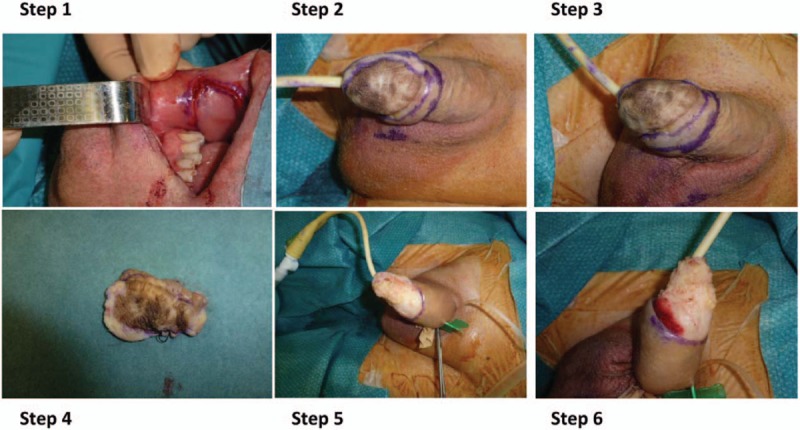
The steps of the surgical excision of the lesion with a healthy margin of 1 cm all over except close to the urethral meatus where it was impossible and where only 5 mm margin was excised.

**Figure 4:**
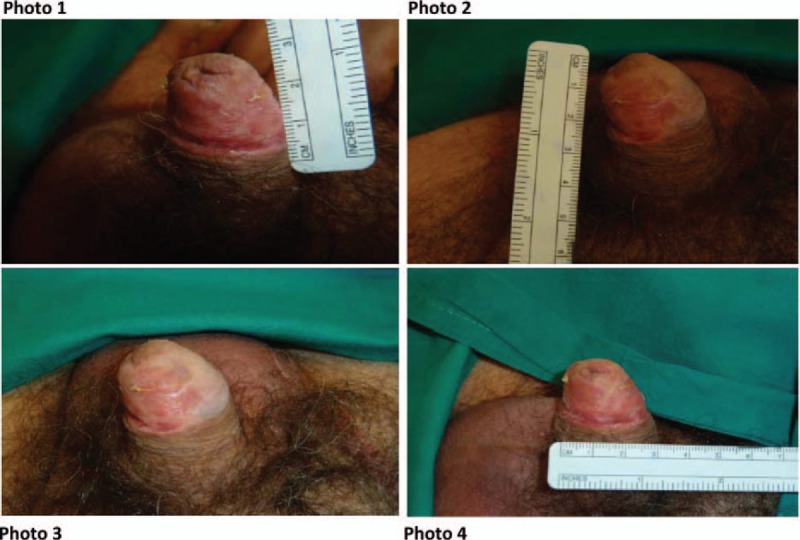
Clinical photographs of patient's penis after 50 months from surgery (Photos 1 and 4: Left side of the glans. Photos 2 and 3: Dorsum of the glans with the left half reconstructed by mucosal graft).

The surgical treatment consisted to excise the lesion with a healthy margin of 1 cm all over except close to the urethral meatus where it was impossible and where only 5 mm of free margin was excised. A full thickness mucosal graft from oral cavity was performed to repair the defect on the glans after the wide excision of MIS (Fig. 3). At the sixth clinical follow-up the patient was alive and disease free at 50 months after surgery (Fig. 4). Moreover, no lower urinary tract symptoms and erectile dysfunction were observed.

## 3 Discussion

Penile melanomas typically present as pigmented macules, papules, or ulcerations with an irregular border on the glans and less often on the prepuce, urethral meatus, and shaft.^[3,7,8]^ A problem in clinical practice is to recognize a pigmented penile lesion as a melanoma. The diagnosis must be confirmed histologically by biopsy of the lesion.^[7]^ The American Joint Committee on Cancer (AJCC) staging protocol for melanoma is the most widely accepted, using the tumor, node, metastasis (or TNM) classification to describe the extent of disease.^[6,9,10]^ The patient presented in this case report was assessed as having AJCC stage 0 (TiS M0 N0) melanoma of the penis.

Surgical approach is the primary treatment of melanoma of the penis and urethra. The main area of controversy of treatment lies with the extent of surgery for localized disease. Recommendations have ranged from wide local excision^[2,11]^ to partial^[2,12,13]^ or total penectomy with bilateral radical groin dissection.^[14,15]^ Furthermore the prognosis of mucosal melanoma is more unfavorable than that of the skin melanoma, so the excision is usually wider.^[16]^ In 2005 according to their experience, literature data and cutaneous melanoma recommendations, Sánchez-Ortiz et al^[17]^ suggested circumcision for lesions located on the foreskin, partial penectomy for lesions located on the glans alone, and partial or radical penectomy for lesions located on the shaft and glans.^[3,17]^

The recommendations for treatment of the MIS penis, due its rarity,^[5,6]^ are very poor and variable.^[1]^ Eight papers are the only reports of this entity in English and German literature (Table 1).^[1,4,5,18–22]^ In 1976, Paul^[18]^ reported a case of lentigo maligna melanoma on glans penis, that some pathologists considered to be a MIS, a noninvasive skin growth.^[23]^ MIS of the penis was described for the first time in 1984 by Begun et al^[19]^ in a 31-year-old male. Demitsu et al^[4]^ reported a case of MIS on the penile shaft treated by a surgical excision with a 0.5 cm margin. Betti et al^[20]^ treated a MIS of penis with subtotal amputation of the glans, while Bechara et al^[1]^ by a local excision with adequate safety margin of approximately 2 cm. Lai et al^[21]^ described a MIS of penis in a man that was a naturist and worked as an electrical tester.^[24]^ The patient was subjected to a fully excision of MIS, because no invasive component was present and the lesion did not extend to the foreskin or lateral margins.^[21]^ Both Napolitano et al^[22]^ and Scalvenzi et al^[5]^ presented a case of patient with a MIS of the glans penis treated successfully using Imiquimod.

**Table 1:**
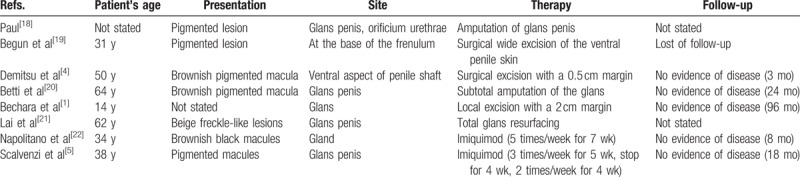
Total of 8 previously reported penis melanoma in situ cases in English and German literature.

In Italy, recent National guidelines for cutaneous melanoma diagnosis and treatment suggest that safety surgical margins for the MIS radicalization are 0.5 cm (recommendation strength = III B).^[25]^

In July 2015, in the United Kingdom the National Institute for Health and Care Excellence (NICE) updated its guideline on assessment and management of melanoma and recommended to consider a clinical margin of at least 0.5 cm when excising stage 0 melanoma and to discuss further management with the multidisciplinary team if excision for stage 0 melanoma does not achieve an adequate histological margin.^[26]^

Considering the scarce literature on MIS penis treatment (from lesion excision^[1,4,19]^ to subtotal^[20]^ or total amputation^[18]^ of the glans) and prognosis and taking in account the last guidelines,^[25,26]^ the authors decided to follow the recommendations for the treatment of MIS located in other sites and, at the same time, to avoid the amputation of the penis for aesthetic reasons and functional impairment. Keeping in mind the worst prognosis of mucosal melanoma they performed surgical excision of the MIS on the patient's penis with 1 cm of normal visible margin all over except close to the urethral meatus where it was impossible and where only 0.5 mm margin was excised.

Mohs micrographic surgery was not taken in consideration in this context on the basis of the current opinion that it is the best option for patients with larger or clinically indistinct lesions.^[27]^

Regards to prognosis, Begun et al^[19]^ reported that 24 of 56 patients (43%) with penile melanoma had inguinal node enlargement at presentation. Most patients die within a few years.^[15,28]^ It seems reasonable that the delay of the diagnosis leads to the poor prognosis.^[4]^ In this case report the patient was alive after 50 months from the chosen excision surgical procedure without recurrence, metastases, and functional disorders.

## 4 Conclusions

MIS of the penis is very rare and recommendations on its treatment are poor in terms of options (medical or surgical) and of adequate surgical margins. This case report highlights the successful of a conservative surgical treatment according to the recent guidelines for the excision of MIS in other sites adding a greater security of 1 cm margin to avoid recurrences and, at the sometime, preserving the aesthetics and function of the penis.
